# Cell–Cell Fusion and the Roads to Novel Properties of Tumor Hybrid Cells

**DOI:** 10.3390/cells10061465

**Published:** 2021-06-11

**Authors:** Mareike Sieler, Julian Weiler, Thomas Dittmar

**Affiliations:** Institute of Immunology, Center for Biomedical Education and Research (ZBAF), Witten/Herdecke University, 58448 Witten, Germany; mareike.sieler@uni-wh.de (M.S.); julian.weiler@uni-wh.de (J.W.)

**Keywords:** cell–cell fusion, cancer, metastasis, drug resistance

## Abstract

The phenomenon of cancer cell–cell fusion is commonly associated with the origin of more malignant tumor cells exhibiting novel properties, such as increased drug resistance or an enhanced metastatic capacity. However, the whole process of cell–cell fusion is still not well understood and seems to be rather inefficient since only a certain number of (cancer) cells are capable of fusing and only a rather small population of fused tumor hybrids will survive at all. The low survivability of tumor hybrids is attributed to post-fusion processes, which are characterized by the random segregation of mixed parental chromosomes, the induction of aneuploidy and further random chromosomal aberrations and genetic/epigenetic alterations in daughter cells. As post-fusion processes also run in a unique manner in surviving tumor hybrids, the occurrence of novel properties could thus also be a random event, whereby it might be speculated that the tumor microenvironment and its spatial habitats could direct evolving tumor hybrids towards a specific phenotype.

## 1. Introduction

In addition to physiological processes, such as fertilization, placentation, myogenesis, osteoclastogenesis and wound healing/tissue regeneration (for review see: [1,2,3,4,5,6,7,8,9]) the biological phenomenon of cell-cell fusion also plays a role in cancer (for review see [5,10,11,12,13,14]), which was already postulated by the German physician Otto Aichel in 1911 [15]. Indeed, cancer cells could either fuse with other cancer cells [16,17,18,19] or could hybridize with macrophages, fibroblasts and stem cells [20,21,22,23,24,25,26,27,28,29,30,31,32,33,34,35,36,37,38,39,40], thereby giving rise to tumor hybrid cells that could exhibit novel properties, such as an increased drug resistance and/or an enhanced metastasis formation capacity. Moreover, within the past two decades, an increasing number of sophisticated studies have been published indicating that cell–cell fusion events really occur in human cancers and that tumor hybrid cells could be detected in both the circulation and metastases [20,22,41,42,43,44,45,46], supporting the hypothesis that cell-cell fusion could give rise to tumor hybrids exhibiting a more malignant phenotype.

However, cell-cell fusion is still a not fully understood process, which not only belongs to those factors/conditions that favor the merging of two (and more) cells, but also to the processes that occur after hybridization, such as heterokaryon-to-synkaryon transition (HST)/ploidy reduction (PR) [47,48,49] and the post-hybrid selection process (PHSP) [30,50]/autocatalytic karyotypic evolution (AKE) [51]. Briefly, these processes describe the merging and random segregation of parental chromosomes to daughter cells (HST/PR), which is associated with the induction of aneuploidy and the subsequent karyotype fine-tuning phase, which is further correlated to losses and gains of whole chromosomes, additional DNA damages, epigenetic alterations and an overall genomic/epigenomic instability (PHSP). All these processes run in a unique manner in evolving tumor hybrids, indicating that the entire process resembles Darwinian evolution since only those hybridized cells with the best fitted genomic/epigenomic background will survive. Whether such tumor hybrids will ultimately exhibit novel properties is not clear since evolution is not a directed, but rather an open process. However, due to the random merging of parental chromosomes concomitant with the induction of aneuploidy/genomic instability, cell-cell fusion will give rise to highly diverse individual tumor hybrids, suggesting that the probability that tumor hybrids could exhibit novel properties might be related to the total number of individual tumor hybrids. The higher the number of unique tumor hybrids is, the higher the probability of tumor hybrids with novel properties would be. This correlation sounds simple, but it is much more complex, since the total number of tumor hybrids (including those with novel properties) depends on several parameters, such as the fusion frequency within the tumor tissue and the survival rates of evolving tumor hybrids. Likewise, it is not clear how many tumor hybrids with novel properties are ultimately required for tumor progression. Is a small number of metastatic tumor hybrids sufficient to induce secondary lesions or does this process depend on a large amount of tumor hybrids exhibiting metastatic properties?

In the present review, we will give a brief overview about the current knowledge of cell–cell fusion in human cancers and will try to give answers to these questions.

## 2. In Vitro and In Vivo Data Supporting Cell–Cell Fusion in Cancer

Data of Wakeling and colleagues revealed that cancer cells varied markedly between each other regarding their fusogenic capacity and that the fusion frequency of some tumor cells was much higher as compared to polyethylene glycol (PEG)-induced fusion [18]. Likewise, homotypic fusion events were also observed for MDA-MB-231 breast cancer sublines exhibiting differential metastatic spreading capacities [19]. Interestingly, the spontaneous fusion of bone metastatic MDA-MB-231 variants with lung metastatic MDA-MB-231 variants gave rise to hybrids with a dual metastasis organotropism to bone and lung [19], indicating that properties of the parental cells were combined in hybrid cells. In contrast, hybrids derived from luminal and basal-like breast cancer cells possessed a more basal-like phenotype, suggesting that the basal-like trait was generally dominant and led to the epigenetic repression of luminal transcription factors [17].

Most studies focusing on heterotypic cell-cell fusion events have investigated tumor hybrids that were derived from cancer cells and macrophages or stem cells. This might be attributed to the fact that both macrophages and stem cells possess fusogenic capacities [20,21,22,23,24,25,26,27,30,31,32,33,34,35,36,38,39,40,52]. For instance, it is well known that macrophages give rise to multinucleated osteoclasts through hybridization [7,53,54] and that stem cells could restore degenerated tissues by cell-cell fusion [55,56,57,58].

About 20 years ago, Rachkovsky and colleagues generated hybrids from murine macrophages and weakly malignant human Cloundman S91 melanoma cells and demonstrated that most tumor hybrids were more malignant than the parental cancer cell line [33]. In fact, of 35 tumor hybrids tested, most were more aggressive and produced metastases sooner and in more mice [33]. However, a striking characteristic was heterogeneity amongst hybrids, with some lines producing no metastases, whereas others induced metastases in up to 80% of mice [33]. While these data are in accordance to Otto Aichel’s hypothesis, they also show that cell-cell fusion is an open process and must not necessarily give rise to more malignant tumor hybrid cells. Powell and colleagues used a parabiosis model to demonstrate that bone marrow-derived cells (BMDCs) fused in vivo with transformed intestinal cells [24]. The analysis of small intestine polyps of an APC^Min/+β − Gal+^ mouse that was surgically joined to a GFP mouse revealed markedly high numbers of GFP and β-Gal double-positive hybrid cells, whereby macrophages were identified as the primary fusion partners [24]. Gene expression studies further demonstrated that hybrids exhibited a unique transcriptome comprising of transcripts that were similarly regulated between hybrids and wild-type intestinal epithelium, that were shared between hybrids and macrophages and that were uniquely expressed in hybrids [24]. Moreover, some genes which have been related to metastasis formation were transcriptionally altered in hybrid cells [24], consistent with the hypothesis that tumor hybrid cells could be more metastatic. However, in vivo studies analyzing the impact of cell-cell fusion in a breast cancer context showed that hybridization events between macrophages and spontaneous *neu+* mammary cancer cells occurred, but without an increased metastatic capacity of tumor hybrids [32]. The co-cultivation of human MCF-7 breast cancer cells and human M2-macrophages gave rise to spontaneously formed tumor hybrids, which showed phenotypic and genetic traits from both parental cells [26]. Moreover, tumor hybrids were positive for the expression of the macrophage marker CD163, which has also been found in human breast cancer specimens [26], suggesting that such cells were originated through cell-cell fusion. Notably, more than 50% CD163-positive breast cancer cells were found in about 26% of the biopsies [26]. Former studies of Shabo and colleagues already demonstrated that breast cancer expression of CD163 was correlated to early distant recurrence and reduced patient survival [59], which further indicates that tumor hybrid cells could be more malignant than parental type tumor cells. Likewise, Gast and colleagues also demonstrated that macrophages could fuse with cancer cells both in vitro and in vivo [22]. Moreover, the authors were able to record a macrophage × tumor cell fusion event concomitant with subsequent cell division by time-lapse video microscopy and were able to identify tumor hybrids not only in the primary tumor, but also in the circulation [22], which further substantiates the hypothesis that tumor hybrids could be the seeds for metastatic lesions.

In addition to macrophages, various studies revealed that tumor hybrids could also originate from fusion events between cancer cells and mesenchymal stem/stromal cells (MSCs) or stem-like cells, which may give rise to tumor hybrid cells exhibiting a cancer stem/initiating cell (CS/IC) phenotype [21,27,52,60,61,62,63,64]. PEG-generated MSC × lung cancer hybrids showed an enhanced metastatic capacity and exhibited an epithelial–mesenchymal transition (EMT) change with the downregulation of E-cadherin and upregulation of N-cadherin, vimentin, α-SMA and fibronectin1 [27]. Moreover, hybrids exhibited increased expression levels of the stem cell transcription factors Oct4, Sox2, Nanog, Kif4 as well as Bmi1, suggesting that they may have acquired stem cell-like properties [27]. Similar data were presented by Dörnen et al. demonstrating that the fusion of MSCs and breast cancer cells led to the formation of tumor hybrids exhibiting diverse and individual (stem cell) characteristics [40]. In vitro and in vivo studies of Melzer and colleagues showed that human MSCs could fuse with human breast cancer cells and that tumor hybrids possessed an enhanced metastatic capacity to distant organs in a much shorter period of time than the parental breast cancer cells [60,61,65]. Even though the frequency of MSC × breast cancer cell fusion events in vivo was only about 0.35% [61], these findings indicate that MSC × tumor cell hybridization could really occur in a living object. These data are in agreement with co-grafting studies using mouse bone marrow MSCs and RM1 murine prostate cancer cells that promoted tumor growth by cell-cell fusion in vivo [36].

## 3. Human In Vivo Data Supporting the Cell–cell Fusion in Cancer Hypothesis

In vitro data clearly indicate that cancer cells could fuse with other cells, thereby giving rise to tumor hybrids. In this connection, a long-lasting question was whether cancer cell-cell fusion events really occur in human cancers and whether such human tumor hybrids really contribute to tumor progression. However, in contrast to animal studies using transgenic mice strains and genetically modified tumor cells, the search for tumor hybrids in human cancers is more difficult and depends on suitable fusion markers. Moreover, tumor hybrids must be clearly distinguished from binucleated cells that may have been formed due to cytokinesis errors, endoreduplication or entosis [66,67].

Tumor hybrid cells in human cancers are commonly detected by the expression of foreign antigens, such as macrophage or hematopoietic lineage epitopes [22,25,59,68,69], the existence of tumor DNA in non-cancer cells [70,71,72] or overlapping genomic markers in cancer patients with a former bone marrow transplantation (BMT) history [22,41,42,43,44] (Table 1).

Macrophage epitopes have been identified in both human breast cancer and colorectal cancer cells, which was associated with early metastasis formation, early distant recurrences and a reduced survival time [59,68,69]. Likewise, the expression of the macrophage marker DAP12 in breast cancer cells was identified in about 66% of breast cancer samples, which was associated with high tumor grade, liver and skeletal metastases and an overall poorer prognosis [69]. Moreover, 16.4% to 23.9% of so-called hemato-epithelial cells, expressing both hematopoietic and epithelial cancer biomarkers, were found in the ascites of epithelial ovarian cancer patients [25]. These findings indicate that putative tumor hybrids can be found in human cancers and that such hybridized cells could contribute to tumor progression. However, genomic instability could also explain the expression of macrophage or hematopoietic lineage markers in cancer cells.

More sophisticated and reliable data indicating the existence of tumor hybrid cells in human cancers were obtained from cancer patients with a former BMT history. Thereby, tumor hybrids were either identified via the presence of the Y chromosome in female cancer patients [22,44] or the genomic overlap of donor and recipient alleles [42,43]. In this connection, Gast and colleagues found out that Y chromosome-positive pancreatic ductal adeno carcinoma (PDAC) cells were not only found in the primary, but also in the circulation of PDAC patients [22]. Moreover, high levels of tumor hybrid cells in the peripheral blood of PDAC patients was further correlated with advanced disease and a statistically significantly increased risk of death [22]. As conventionally defined circulating tumor cells were not correlated with stage or survival and were detected at quantities an order of magnitude lower than circulating tumor hybrids in metastatic PDAC disease [22], these findings strongly indicate that tumor hybrids really could originate in human cancers and could contribute to disease progression. However, it has to be taken into account that the presence of the Y chromosome could also be attributed to the so-called phenomenon of fetal cell microchimerism (FCM), which is the persistence of fetal cells within the mother for decades after pregnancy without any apparent rejection [76]. Indeed, FCM cells have been found in a variety of human cancers, such as breast, cervix, lung and melanoma and displayed epithelial, hematopoietic, mesenchymal and endothelial lineage differentiation [76]. Whether FCM cells could undergo malignant transformation remains unclear, which also applies to the possibility that FCM cells could fuse with tumor cells. The best proof for real tumor hybrid cells in human cancers was provided by Lazova et al. and LaBerge and colleagues [42,43]. In both studies, single tumor cells of melanoma patients with a former BMT history were analyzed by short tandem repeat (STR) analysis and revealed an overlap of multiple donor and recipient alleles [42,43]. In the study of Lazova et al., overlapping donor and recipient alleles were found in a melanoma brain metastasis derived from an unknown primary tumor [43], whereas in the work of LaBerge and colleagues, tumor hybrids with overlapping alleles were identified in the primary melanoma and a lymph node metastasis [42]. In fact, these two studies clearly indicate that human BMDCs can fuse with human cancer cells, thereby giving rise to tumor hybrid cells that could exhibit metastatic properties.

## 4. How Do Cancer Cells Fuse?

Even though it is known that cancer cells, like other cells, possess fusogenic capacities, it still remains to be elucidated how cell–cell fusion is directed. First of all, cell-cell fusion is a complex and tightly regulated process. Cells are not per se fusogenic, but have to enter a pro-fusogenic state first [1,4,6,7,9,77]. Likewise, hybridized cells have to return into a non-fusogenic state to prevent further uncontrolled fusion events.

It is well known that cell-cell fusion is facilitated by so-called fusogens, which bring the lipid bilayers of two cells into immediate contact, catalyze the formation of energy-intensive fusion intermediates and open the fusion pore [9]. However, only a handful of fusogens have been identified so far in humans, which usually direct physiological cell–cell fusion processes, such as syncytins in placentation, myomaker and myomerger in myogenesis, and IZUMO and Juno in fertilization [78,79,80,81,82,83,84,85]. Several studies revealed that syncytins were also involved in tumor cell-cell fusion events [86,87,88,89]. For instance, Bjerregard and colleagues demonstrated that breast cancer endothelial cell fusion events were mediated by syncytin-1 [86], which, surprisingly, was associated with a better prognosis for afflicted patients [90]. In contrast, the overexpression of syncytin-1 in urothelial cell carcinoma of the bladder has been proposed as an indicator for urothelial cell carcinoma risk since it was associated with proliferation, viability and an increased fusion frequency [89]. Likewise, proliferation and cell–cell fusion of endometrial carcinoma were induced by syncytin-1 [87]. Moreover, the authors further demonstrated that the syncytin-1-mediated fusion of endometrial cancer cells was inversely correlated to transforming growth factor-β (TGF-β) levels. Thereby, high TGF-β levels inhibited cell–cell fusion, but induced cell proliferation, whereas blocking of TGF-β was associated with an enhanced frequency of cell–cell fusion events [87]. The finding that the cell–cell fusion of endometrial cancer cells is regulated by TGF-β is in agreement to the hypothesis that cell-cell fusion is a tightly controlled process. In this connection, Yan and colleagues also showed that the Wnt/β-catenin-mediated up-regulation of syncytin-1 expression contributed to the tumor necrosis factor-α (TNF-α)-enhanced fusion of human umbilical vein endothelial cells (HUVECs) and oral squamous cell carcinoma (OSCC) cells [88]. While these data further substantiate the idea that cell-cell fusion is a tightly regulated process, they also indicate that cell-cell fusion could be induced. Indeed, TNF-α or inflammatory conditions in general have been associated with an enhanced cell-cell fusion frequency [60,91,92,93,94,95,96,97]. However, it remains to be elucidated how TNF-α or inflammatory conditions ultimately induce cell-cell fusion and/or support/induce the conversion of cells from a non-fusogenic into a fusogenic state. In this connection, we have recently demonstrated that the TNF-α induced fusion of human M13SV1-Cre breast epithelial cells with human MDA-MB-435-pFDR1 cancer cells was attributed to matrix metalloproteinase 9 (MMP9) expression [95]. Both the blockade of MMP9 activity using a specific inhibitor [95] and the inhibition of MMP9 expression by minocycline [94] markedly impaired the TNF-α-induced cell-cell fusion rate. Interestingly, a similar mechanism has also been described for macrophages [96], but it remains completely unclear how MMP9 is involved in the entire cell-cell fusion process. It might be possible that proteolytic enzymes such as MMPs or ADAMs may reduce the overall distance between both fusion partners by degrading cell adhesion molecules, mobilize and activate masked growth factors embedded in the extracellular matrix or facilitating cell-cell fusion by fostering cell–cell interactions [98,99,100]. In addition to MMP9 or proteases in general, several other proteins/molecules, such as cell adhesion molecules, cytokines, chemokines, receptors, actin-modifying enzymes and lipids have been identified that are all involved in cell–cell fusion (for review see: [1,4,6,7,8,9]), which substantiate the complexity of this process.

It is well known that the tumor microenvironment resembles chronically inflamed tissue [101,102,103] and because of that, tumors have been proposed as wounds that do not heal [104]. As inflammation/inflammatory conditions induce cell-cell fusion, it might be speculated that the chronically inflamed tumor microenvironment provides a fusion-friendly milieu. In any case, the number of cell–cell fusion events within the tumor microenvironment remains ambiguous. Indeed, animal studies have shown that the intratumoral cancer cell–cell fusion frequency could be between 0.0066% to 6% [16,22,61,105,106], but these estimated counts are only valid for those tumor hybrids that have been detected due to the co-expression of specific fusion markers. Homotypic tumor cell-cell fusion events or tumor hybrids that have lost the expression of the fusion markers will not be considered as hybrids in the evaluation. Such indistinguishable or invisible tumor hybrids have also been referred to as dark matter hybrids, which could contribute to tumor growth and progression; albeit they cannot yet be detected and quantified [13]. Likewise, cancer cell lines differed markedly between each other regarding their fusogenic capacities [16,18], indicating that highly fusogenic and less fusogenic cancer cell lines exist, which could also have an impact on the frequency of cell–cell fusion events. Similar findings were also presented for cells of the hematopoietic lineage, such as macrophages, B-lymphocytes and T-lymphocytes, which were all capable of fusing with transformed intestinal cells [24]. However, in comparison to macrophages, which were the main fusion partners (about 20%) the fusogenic capacity of B- and T-lymphocytes was rather low (about 3%) [24]. Moreover, the frequency of cell–cell fusion events detected in vivo likely depends on additional parameters, such as the used cancer cell lines (human vs. murine origin), the site of implantation (orthotropically vs. non-orthotropically), the addition of matrix components and cells (which have an impact on tumor cell engraftment) and the used mouse strain (immune compromised mice strains: BALB/c nude, non-obese diabetic/severe combined immunodeficiency (NOD/SCID) vs. non-immunocompromised (transgenic) mice strains). All these parameters could have an impact on the total number of cell–cell fusion events in animal tumor models. A small tumor size consisting of less fusogenic cancer cells would result in a low frequency of cell–cell fusion, whereas the rate of tumor hybrids would be higher in large tumors comprising of highly fusogenic cancer cells. Immunocompromised mice strains are routinely used for tumorigenic studies using human cancer cell lines, but they lack B- and T-lymphocytes, which exhibit a certain degree of fusogenecity. It is known that human cancer cells could fuse with murine macrophages and stromal cells [33,37,107], but it remains unclear whether the frequency of human × mouse cell–cell fusion events is comparable to the frequency of human cell–cell fusion events.

Whether the cell–cell fusion frequency in human cancers could be as high as observed in animal studies remains to be elucidated. As stated above, only visible tumor hybrid cells can be detected so far, whereas invisible tumor hybrids remain undetected. Likewise, the total rate of cell–cell fusion events depends on several parameters, such as the overall fusogenecity of cancer cells and normal cells and cell-cell fusion influencing factors/conditions, which could vary between different types of cancer. Moreover, the cell–cell fusion frequency number only indicates the rate of cell–cell fusion events that have been determined at a certain time point, but it neither represents the total number of surviving tumor hybrids nor whether tumor hybrids possess novel properties. Both depend on the outcome of post-fusion processes.

## 5. Post-Fusion Processes

Most studies indicate that tumor hybrids are more aggressive and more malignant than their parental cancer cells [16,17,18,19,20,21,22,23,24,25,26,27,28,29,30,31,32,33,34,35,36,37,38,39,40,42,43], suggesting that cell–cell fusion might be a directed process which per se converts low-malignant tumor cells into highly malignant cancer cells through hybridization with other cells. This suggestion, however, is not correct, since the ultimate phenotype of tumor hybrids cannot be predicted, which is attributed to post-fusion processes, such as HST/PR [47,48,49] and PHSP/AKE [30,50,51] that run in a unique manner in each evolved tumor hybrid cell.

**Figure 1:**
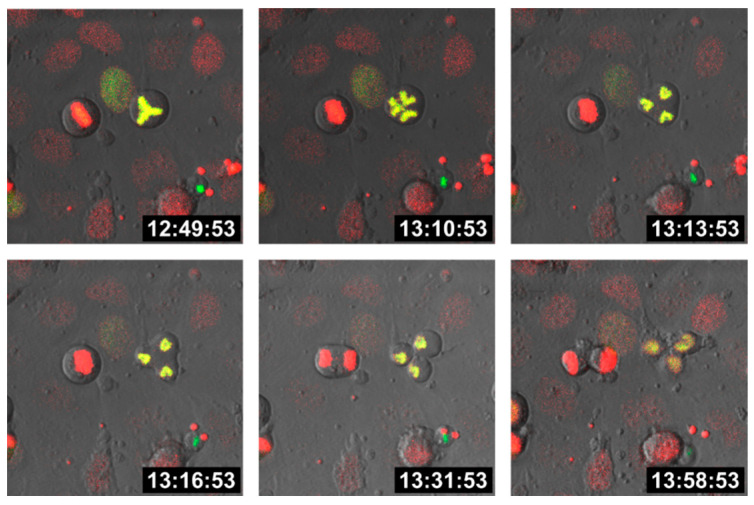
Tripolar division of a hybrid cell. M13SV1 human breast epithelial cells [108] were either stably transduced with pH2B-GFP (kind gift from Geoff Wahl; Addgene plasmid #11680; http://n2t.net/addgene:11680; RRID: Addgene_11680; accessed on 15 April 2021) or pH2B_mCherry_IRES_puro2 (kind gift from Daniel Gerlich; Addgene plasmid #21045; http://n2t.net/addgene:21045; accessed on 15 April 2021, RRID:Addgene_21045). Hybrid cells were cultured on chamber slides (ThermoFisher Scientific GmbH, Schwerte, Germany) and time-lapse series were recorded using a Leica TCS SP5 confocal laser scanning microscope (Leica, Wetzlar, Germany).

Cell fusion first results in bi- (or multi-) nucleated cells, also termed heterokaryons, which could either remain in this state or could undergo HST/PR [47,48,49], thereby giving rise to daughter cells with one-half chromosomal content. In fact, HST/PR is a complex and not yet fully understood process. HST/PR requires an active cell cycle and the resolution of both nuclear membranes results in the random merging of parental chromosomes, which are subsequently segregated via tripolar and multipolar divisions, thereby giving rise to aneuploid daughter cells [48,49] (Figure 1).

Moreover, HST/PR is further associated with additional chromosomal aberrations, such as double strand breaks and chromothripsis [48,49,109,110]. Indeed, chromosome missegregation, particularly when coupled with breakage, is often followed by irreversible cell cycle arrest, impaired proliferation or cell death [111]. This could either be attributed to the lack of crucial chromosomes or due to the induction of apoptosis if tumor cells have fused with normal cells harboring a functional set of tumor suppressor genes. This is in view, findings suggest that breast cancer cell × cancer-associated fibroblast hybrids were not viable, whereas homotypic and heterotypic breast cancer cell hybrids could be generated and propagated [16].

**Figure 2:**
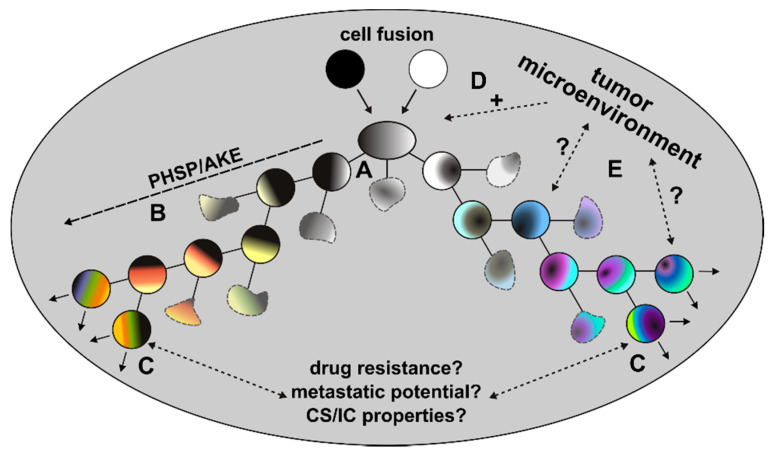
Post-fusion processes. Due to the random segregation of merged parental chromosomes, different viable and non-viable tumor hybrid cells with a unique karyotype originate (A). Surviving tumor hybrids will first undergo PHSP/AKE, which is a fitting process that gives rise to tumor hybrids with a rather stable, but still aneuploid and genomic instable karyotype (B). The initial period of PHSP/AKE might be associated with an increased cell death (blurry cells). Surviving and proliferating tumor hybrids could exhibit novel characteristics, such as an increased drug resistance, metastatic potential or CS/IC properties (C). The role of the tumor microenvironment in these processes remains unclear. While it is assumed that the chronically inflamed tumor microenvironment might positively trigger cell-cell fusion events (D), its role in PHSP/AKE and acquisition of novel properties of tumor hybrids remains unclear (E).

In addition to these initial HST/PR dependent events, the genomic background of tumor hybrids is further fine-tuned during the next divisions to give rise to stable proliferating tumor hybrids. This fine-tuning process, which has also been named the post-hybrid selection process (PHSP) [30,50], resembles to the so-called autocatalytic karyotypic evolution (AKE) model that was proposed for aneuploid cells [51] and which is associated with additional gains and losses of whole chromosomes, further chromosomal aberrations and an overall increased genomic/epigenomic instability (Figure 2).

Like HST/PR, PHSP/AKE also runs in a unique manner in each tumor hybrid cell, thereby giving rise to unique progenies. For instance, Zhao and colleagues demonstrated that the number of chromosomes in fusion-derived clones near triploid or tetraploid at early passage usually decreased with repeated passage [112]. Likewise, a substantial shift in the phenotypes of MDA-MB-231 × SUM159PT hybrids at early (2) and extended (10) passages was observed, which was further consistent with the selection of a fit subpopulation of hybrid cells and additional diversification [16]. Hybrid clones derived from human breast epithelial cells and human breast cancer cells possessed a unique mean chromosomal number [64,113], which is in accordance with recent data of Delespaul et al. and Lartigue and colleagues who also showed that mesenchymal tumor hybrid clones exhibited a unique chromosomal content [114,115,116]. In this connection, it should be kept in mind that PSHP/AKE does not stop at a certain time point, e.g., after 10 or 15 divisions. It is a continuous process, whereby it might be speculated that it runs much faster during early passages and much slower during late passages once a relative stable karyotype has been established in tumor hybrids. Nonetheless, aneuploid/genomic/epigenomic instable tumor hybrid cells will always give rise to aneuploid/genomic/epigenomic instable daughter hybrids, etc., which is definitely associated with an increase in intratumoral heterogeneity.

In vitro studies revealed that the incidence of tumor hybrids surviving both HST/PR and PHSP/AKE could be up to 1% [18,117]. How many tumor hybrids will survive both HST/PR and PHSP/AKE in vivo remains unclear. As mentioned above, only those cells possessing specific fusion markers are considered as tumor hybrids, which excludes homotypic tumor hybrids and cells that have lost marker expression. Likewise, it cannot be ruled out that the specific survival rates of discrete tumor hybrid populations might differ, which would be similar to the unique fusogenic properties of cancer cells and normal cells. For instance, no colonies were derived from 1456 tracked RL-1/HPS-14 cancer–stromal hybrids, whereas 41 colonies were grown from 1456 tracked RL-1/HPS-15 cancer–stromal hybrids [118]. HPS-14 cells represent normal human prostate myofibroblast stromal cells, whereas HPS-15 cells are cancer-associated human prostate myofibroblast stromal cells [118]. Due to the low tumor hybrid survival rate, the authors speculated whether the fusion of cancer cells with stromal cells would be suitable for cancer therapy since the primary fate of cancer stromal hybrids was death [118]. Likewise, different numbers of tumor hybrid clones were derived from human M13SV1-EGFP-Neo breast epithelial cells and human HS578T-Hyg breast cancer cells (M13HS-X; X = 1–5, 7–10), human MDA-MB-435-Hyg cancer cells (M13MDA435-X, X = 1–4) and human MDA-MB-231-Hyg breast cancer cells (M13MDA231-X, X = 1–14) [21,113]. Given that identical cell numbers have been used for co-culture experiments (1 × 10^6^ cells per cell line) these data indicate that the overall survival rate of tumor hybrids in relation to the total number of parental cells was rather low [21,113].

However, it has to be considered that cell-cell fusion is not a one-time event, but rather a continuous process, suggesting that the total number of tumor hybrids should increase steadily with time. Likewise, tumor hybrids are proliferating and give rise to progenies, which will also steadily increase the pool of tumor hybrids within the tumor microenvironment. Moreover, all these calculations and considerations are based on visible tumor hybrids, whereas the unrecognized fraction of invisible tumor hybrids is excluded, suggesting that both the fusion frequency and survival rate could be higher than anticipated. Mathematical models of Miroshnychenko and colleagues demonstrated that the intratumoral heterogeneity is much more driven by both fusion and mutations than mutations alone despite rather low fusion probabilities of 6.6 × 10^−3^ in vitro and 6.6 × 10^−5^ in vivo [16]. Interestingly, higher levels of mutational heterogeneity dramatically enhanced the impact of fusion-mediated recombination, which should therefore be more pronounced in tumors with higher levels of mutational clonal heterogeneity [16]. Moreover, cell-cell fusion-mediated diversity was acquired much faster and higher in 3D than in 2D contexts, which is likely attributed to the higher number of genetically distinct neighbors [16]. Hence, even a rather low fusion (and survival) probability could have a huge impact on intratumoral heterogeneity and tumor progression.

## 6. Novel Properties of Tumor Hybrids

Cell fusion is an open process, which means that it could not be predicted whether tumor cells will be viable or non-viable and whether they could exhibit novel properties or not. Hence, the fraction of viable tumor hybrids will be a mixture of diverse hybrid clones exhibiting individual properties, like a rainbow consisting of different colors. Some hybrid clones could be more sensitive to cancer therapy, whereas other hybrid clones could be highly drug-resistant. Likewise, some hybrids could be non-metastatic, whereas other hybrid clones could be highly metastatic. It is also possible that individual hybrid clones could be both more metastatic and drug-resistant, whereas other hybrids are either metastatic or drug-resistant, and some could be neither metastatic nor drug-resistant. Finally, some tumor hybrid clones could be highly proliferative, whereas others rather remain in a quiescent state. In fact, some studies demonstrated that cell-cell fusion could also give rise to tumor hybrid cells exhibiting a weaker or even no metastatic capacity as compared to the parental cancer cell line. This, for instance, was demonstrated by Rachkovsky and colleagues [33]. While the majority of macrophage × melanoma hybrids were more aggressive and produced metastases sooner and in more mice, the metastatic capacity of some tumor hybrids was lower or even zero in comparison to the parental cancer cell line [33]. Fahlbusch et al. have recently shown that hybrid cells that were derived from human breast epithelial cells exhibiting stem cell properties and human breast cancer cells varied markedly regarding their capacities to form mammospheres and the amount of ALDH1-positive cells [113]. Tumor hybrids that were derived from murine *neu+* mammary cancer and mouse macrophages exhibited no increased metastatic potential [32].

How many more malignant tumor hybrid clones will evolve in comparison to all tumor hybrid clones remains ambiguous. Nonetheless, it can be assumed that the probability that surviving tumor hybrid clones with novel properties will originate is directly correlated to the total number of evolved tumor hybrid clones. This assumption is supported by mathematical data revealing that despite a low fusion rate, the diversity and clonal richness is much more driven by both fusion and mutations [16]. Moreover, the impact of cell-cell fusion on the diversification of individual tumor hybrid clones was substantially higher in a 3D environment due to higher numbers of genetically distinct neighbor cells [16], suggesting that tumor hybrids exhibiting novel properties might originate from heterotypic rather than from homotypic cell–cell fusion events.

These data further support the importance of the chronically inflamed tumor microenvironment, which may not only provide a fusion-friendly milieu, but also provides a high number of different fusion partners. The chronically inflamed tumor microenvironment represents a complex mixture of tumor cells, fibroblasts, immune cells, MSCs, endothelial cells, neurons, extracellular matrix components, cytokines, chemokines, growth factors, proteases, hormones and neurotransmitters, which all physically and chemically interact with each other [50,119,120,121]. Likewise, the chronically inflamed tumor microenvironment plays a crucial role in directing various processes, such as EMT, trans- or retrodifferentiation, autophagy, metastases formation and epigenetic alterations [50,119,120,121] and putatively even PHSP [50]. Whether the chronically inflamed tumor microenvironment might provide survival signals ensuring that tumor hybrids will successfully pass through this karyotype fine-tuning process or whether it might have an impact on the final phenotype of tumor hybrids is not clear but cannot be ruled out. The chronically inflamed tumor microenvironment consists of different spatial habitats, each harboring a specific set of cellular and environmental conditions, which all closely interact with and influence each other [122,123,124]. Hence, the cellular composition has an impact on the composition of the environment and vice versa, which additionally drives intratumoral heterogeneity in a Darwinian manner [122,123,124]. With regard to cell–cell fusion, this would mean that the specific spatial habitat in which tumor hybrids have formed could also have an impact on the cells’ phenotype, since evolving tumor hybrids have to adopt to the specific spatial environment in order to survive. It is commonly accepted that CS/ICs reside in a specialized compartment of the tumor microenvironment termed niche [125]. Fusion between cancer cells and MSCs or stem-like cells could give rise to tumor hybrid cells exhibiting a CS/IC phenotype [21,27,52,60,61,62,63,64], suggesting that the origin of tumor hybrids close to this specialized compartment might give rise to CS/IC-like tumor hybrids. This would mean that the ultimate phenotype of tumor hybrids is not purely random, but could be driven to a certain phenotype dependent on the specific spatial tumor microenvironment.

Intratumoral heterogeneity is a hallmark of cancer [126] and the main reason for acquired resistance during therapy, which enables some tumor cells to survive treatment and facilitates the development of new therapy-resistant phenotypes [124]. Given that cell–cell fusion together with mutations is a potent driver of diversification, clonal richness and an overall enhanced tumoral heterogeneity [16], it can be concluded that the process of cell-cell fusion is involved in the origin of therapy-resistant phenotypes, which is in line with the hypothesis that cell–cell fusion could give rise to tumor hybrids exhibiting novel properties, such as an enhanced drug resistance [10,127,128,129,130]. Hence, the process of cell–cell fusion might be a suitable target for novel anti-cancer therapies. The inhibition of homotypic and heterotypic cell–cell fusion events should be correlated with a decreased diversification and clonal richness, and an overall reduced intratumoral heterogeneity.

While this conclusion sounds conclusive, the reality is much more complex. To understand the process of cell–cell fusion better and its impact on tumor progression, it is mandatory to clarify how many tumor hybrids will reside in the primary tumor, in the circulation and in metastases. This, however, requires appropriate valid fusion or hybrid cell markers. Cell-cell fusion could give rise to binucleated cells, but also cytokinesis errors, endoreduplication or entosis [66,67]. The expression of hematopoietic lineage markers could be related to a former cell-cell fusion event [22,25,59,68,69], but to genomic instability as well. Thus far, tumor hybrids have been only clearly identified by genetic markers in cancer patients with a former BMT history [22,41,42,43,44], but these cases are pretty rare. Mathematical data suggesting that even a low fusion probability could have a pronounced effect on intratumoral heterogeneity [16] are promising, but need to be validated.

Likewise, cell-cell fusion is still a not well-understood process, which applies to both proteins and conditions that regulate and induce the merging of two (and more) cells. Do cancer cells fuse in a uniform manner through a conserved mechanism or does the fusion machinery differ between different types of cancer? Without this knowledge, the development of appropriate therapies would not be possible. Moreover, the correlation between cell–cell fusion, an enhanced intratumoral heterogeneity and therapy resistance suggests that therapy-resistant cancer cells predominantly originate from hybridization events. However, therapy-resistant cancer cells could also originate due to genetic and epigenetic alterations or interactions with the microenvironment without fusion [124], which is another point that has be clarified before specific therapies could be developed.

## 7. Conclusions

Within the past two decades, the view on cell-cell fusion in cancer has markedly changed. This fact is not only attributed to more sophisticated animal models allowing one to visualize and characterize the biology of tumor hybrids in vivo, but also to studies indicating that tumor hybrids were found in human cancers [22,24,41,42,43,44,45]. Moreover, mathematical models revealed that even a rather low fusion rate could have a pronounced effect of diversification and clonal richness, thereby enhancing intratumoral heterogeneity [16].

However, despite these promising data which demand to be validated in ongoing studies, cell–cell fusion is still a not well-understood process, which not only applies to the proteins/conditions that favor (tumor) cell-cell fusion, but also to the fate of evolved tumor hybrid cells. Whether tumor hybrids will survive or not and whether they will possess novel properties is purely random. It might be assumed that the tumor microenvironment could have an impact on evolving tumor hybrids, such as providing survival signals or other factors directing the cells towards a certain phenotype, but this is less clear and purely speculative.

A general problem is the lack of appropriate fusion and hybrid markers, which clearly identify tumor hybrids. Current strategies are primarily focusing on heterotypic tumor hybrids exhibiting specific markers of both parental cells, such as the co-expression of epithelial and hematopoietic markers or overlap of donor and recipient alleles. This strategy, however, only works on “visible” tumor hybrids that possess both parental markers. Tumor hybrids which have lost one marker or homotypic tumor hybrids will not be categorized as hybrids and remain invisible.

To further understand the process of cell–cell fusion in cancer, which would be a prerequisite for putative novel therapies in the future, it is mandatory to investigate which proteins and conditions are regulating and inducing the hybridization of two cells. Likewise, novel fusion and hybrid markers have to be identified to clarify whether tumor hybrids are a common or rather a minor population in human cancers.

## Figures and Tables

**Table 1:** Evidence for cell–cell fusion in human cancers.

Tumor Type	Normal Cells	Marker	Reference
breast cancer	macrophages	CD163, MAC387, DAP12	[59,69,73]
colon/colorectal cancer	macrophages	CD163, MAC387, DAP12	[68,73]
		CD14, CD45, cytokeratin	[20]
epithelial ovarian cancer	BMDCs	CD45, CXCR4	[25]
melanoma	macrophages	CD14, CD45, cytokeratin	[20]
	stromal cells	BRAF (V600E) mutation	[72]
	BMDCs	STR-analysis *	[42,43]
multiple myeloma	osteoclasts	myeloma specific translocations	[70,71,74]
non-small cell lung cancer	macrophages	CD14, CD45, cytokeratin, EpCAM	[46]
pancreatic ductal adenocarcinoma	macrophages	CD45, cytokeratin	[45,75]
	BMDCs	CD45, EpCAM, Y chromosome *	[22]
renal cell carcinoma	BMDCs	blood group alleles *, Y-chromosome *	[41,44]

* Cancer patients with a BMT history.

## Data Availability

Not applicable.

## Abbreviations

ADAMs	a disintegrin and metalloproteases
AKE	autocatalytic karyotypic evolution
BMDCs	bone marrow-derived cells
BMT	bone marrow transplantation
CS/IC	cancer stem/initiating cell
E/M	epithelial/mesenchymal
EMT	epithelial-mesenchymal transition
EpCAM	epithelial cell adhesion molecule
FCM	fetal cell microchimerism
HST	heterokaryon-to-synkaryon transition
HUVECs	human umbilical vein endothelial cells
MET	mesenchymal epithelial transition
MMP9	matrix metalloproteinase 9
MSCs	mesenchymal stem/stromal cells
NOD/SCID	non-obese diabetic/severe combined immunodeficiency
OSCC	oral squamous cell carcinoma
PDACs	pancreatic ductal adeno carcinoma
PEG	polyethylene glycol
PHSP	post-hybrid selection process
PR	ploidy reduction
TGF-β	transforming growth factor-β
TNF-α	tumor necrosis factor-α
